# Eclectic Department

**Published:** 1856-11

**Authors:** 


For the following valuable resume of matters of practice floating in the medical press, we are indebted to the Memphis Med. Recorder:

*Hemoptysis.*—M. Aran disapproves of blood-letting in this affection, and recommends, particularly in feeble constitutions, the oil of turpentine in doses of ten to thirty drops. Ergot is somewhat less efficacious. Chloride of soda in doses of one to two and a half drachms proves very efficacious. Tannin and gallic acid are recommended, but the latter is preferred because it does not exert the same desiccating effect upon the tissues, or induce the obstinate constipation produced by tannin. In those cases in which veratrine was employed, the bleeding ceased as if by enchantment. He has succeeded also with a combination of digitalis and nitre. The gallic acid and the turpentine are the two remedies most to be relied upon in severe cases, but they may be aided by ligatures to the limbs, and ice to the chest.

*Irrigation in Phagedena.*—A successful experiment has been tried in Guy's Hospital, in the treatment of phagedenic ulcers by constant irrigation. The affected limb is placed on some water-proof material, with a reservoir above the bed supplied with warm water, and by means of an elastic tube a stream is kept constantly flowing over the surface of the sore. The discharge is thus washed away as soon as formed, and the ulcer assumes the clean pale appearance of a piece of soaked flesh. The theory is, that phagedenic action is a process of local contagion—the *materies morbi* by which the ulcer spreads being its own pus. Nitric acid relieves the difficulty less perfectly, and with greater suffering, by decomposing the morbid discharge; but to be effectual, the whole surface of the ulcer must be destroyed to a considerable depth.

*Sweet-Gum Bark*—(*Liquidambar Styraciflua.*)—This bark is extensively used in this region as a tonico astringent in diarrhœa, and generally in the form of a cold infusion, or decoction; but Dr. Wright, of Ky., recommends a sirup made after the formula of that for the preparation of the wild cherry bark, as follows: Take of sweet-gum bark, in coarse powder, five ounces; sugar (refined) two pounds; water a sufficient quantity. Moisten the bark thoroughly with water, let it stand for twenty-four hours in a close vessel, then transfer it to a percolator, and pour water upon it gradually until a pint of filtered liquid is obtained. To this add the sugar in a bottle, and agitate occasionally until it is dissolved. The dose for an adult is about one ounce. This is a very pleasant medicinal sirup for children, and well suited to bowel complaints indicating the use of astringent remedies.

*Extract of Belladonna.*—This extract may be prepared for exhibition to children by dissolving four grains in an ounce of distilled water, from five to fifteen drops of which may be given in sweetened water three or four times a day. Its physiological effects are dryness and redness of the throat, and efflorescence of the skin, by exciting which it is supposed to exercise some preventive or modifying influence in scarlatina. It has been found useful, also, in whooping-cough, and in hoarseness from measles or a protracted catarrh. Probably its efficacy consists mainly in changing the condition of the pharyngeal membrane.

*Borax Enemata.*—It is contended by a medical writer, that two kinds of diarrhœa prevail among children; one, symptomatic of organic disease of the intestinal mucous membrane; the other, an idiopathic affection of the same membrane, having no appreciable lesion. It is in the latter affection that borax enemata are recommended, which are supposed to have an effect on the mucous membrane of the larger intestines analogous to that produced on the mouth in aphthous affections. A solution of four to six drachms of borax in six ounces of water may be used for this purpose. In this country, however, it must be considered that in a large majority of cases of infantile diarrhœa, the local lesion is symptomatic of periodic fever, and cannot be permanently cured without antiperiodic treatment.

*Iron in Erysipelas.*—A French journal states that Velpeau places the greatest reliance in iron as an external remedy founded upon his experience in one thousand cases. He uses the proto-sulphate of iron in solution, about twelve grains to the ounce of water, or eight parts to thirty of lard. The lotion is applied by soft compresses or cloths kept constantly moist. Probably most of these were surgical cases in hospital, requiring less attention to constitutional remedies than the disease generally does in this country. We very much doubt whether this local treatment is to be preferred to that with Lugol's solution of iodine.

